# A Web-Based Mobile App (INTERACCT App) for Adolescents Undergoing Cancer and Hematopoietic Stem Cell Transplantation Aftercare to Improve the Quality of Medical Information for Clinicians: Observational Study

**DOI:** 10.2196/18781

**Published:** 2020-06-30

**Authors:** Anita Lawitschka, Stephanie Buehrer, Dorothea Bauer, Konrad Peters, Marisa Silbernagl, Natalia Zubarovskaya, Barbara Brunmair, Fares Kayali, Helmut Hlavacs, Ruth Mateus-Berr, David Riedl, Gerhard Rumpold, Christina Peters

**Affiliations:** 1 Stem Cell Transplantation-Outpatient and Aftercare Clinic St. Anna Children’s Hospital Medical University Vienna Vienna Austria; 2 Children's Cancer Research Institute Vienna Austria; 3 Department of Clinical and Health Psychology Faculty of Psychology University of Vienna Vienna Austria; 4 Department of Stem Cell Transplantation St. Anna Children's Hospital Medical University of Vienna Vienna Austria; 5 Faculty of Computer Science University of Vienna Vienna Austria; 6 Center for Public Health Medical University Vienna Vienna Austria; 7 Science Communication Children's Cancer Research Institute Vienna Austria; 8 Center for Teacher Education Vienna University of Technology Vienna Austria; 9 Center for Didactics of Art and Interdisciplinary Education University of Applied Arts Vienna Vienna Austria; 10 Department of Psychiatry, Psychotherapy and Psychosomatics University Clinic of Medical Psychology Innsbruck Austria; 11 Evaluation Software Development Innsbruck Austria

**Keywords:** mobile app, adolescents, cancer, stem cell transplant, self-reported heath status, medical information exchange, mobile phone

## Abstract

**Background:**

A growing number of cancer and hematopoietic stem cell transplant (HSCT) survivors require long-term follow-up with optimal communication schemes, and patients' compliance is crucial. Adolescents have various unmet needs. Regarding self-report of symptoms and health status, users of mobile apps showed enhanced compliance. Currently, HSCT aftercare at the HSCT outpatient clinic of the St. Anna Children’s Hospital in Vienna, Austria, is based on handwritten diaries, carrying various disadvantages. Recently, we developed the prototype of a web-based, self-monitoring gamified mobile app tailored for adolescents: the INTERACCT (Integrating Entertainment and Reaction Assessment into Child Cancer Therapy) app.

**Objective:**

This observational, prospective study evaluated the usability of the INTERACCT app for tracking real-time self-reported symptoms and health status data in adolescent HSCT patients and a healthy matched control group. The primary outcome of the study was the quality of the self-reported medical information. We hypothesized that the mobile app would provide superior medical information for the clinicians than would the handwritten diaries.

**Methods:**

Health data were reported via paper diary and mobile app for 5 consecutive days each. The quality of medical information was rated on a 5-point scale independently and blinded by two HSCT clinicians, and the duration of use was evaluated. A total of 52 participant questionnaires were assessed for gaming patterns and device preferences, self-efficacy, users’ satisfaction, acceptability, and suggestions for improvement of the mobile app. Interrater reliability was calculated with the intraclass correlation coefficient, based on a two-way mixed model; one-way repeated-measures analysis of variance and *t* tests were conducted post hoc. Descriptive methods were used for correlation with participants’ demographics. For users’ satisfaction and acceptability of the mobile app, the median and the IQR were calculated.

**Results:**

Data from 42 participants—15 patients and 27 healthy students—with comparable demographics were evaluated. The results of our study indicated a superiority of the quality of self-reported medical data in the INTERACCT app over traditional paper-and-pencil assessment (mobile app: 4.14 points, vs paper-based diary: 3.77 points, *P*=.02). The mobile app outperformed paper-and-pencil assessments mainly among the patients, in particular among patients with treatment-associated complications (mobile app: 4.43 points, vs paper-based diary: 3.73 points, *P*=.01). The mobile app was used significantly longer by adolescents (≥14 years: 4.57 days, vs ≤13 years: 3.14 days, *P*=.03) and females (4.76 days for females vs 2.95 days for males, *P*=.004). This corresponds with a longer duration of use among impaired patients with comorbidities. User satisfaction and acceptability ratings for the mobile app were high across all groups, but adherence to entering a large amount of data decreased over time. Based on our results, we developed a case vignette of the target group.

**Conclusions:**

Our study was the first to show that the quality of patient-reported medical information submitted via the INTERACCT app embedded in a serious game is superior to that submitted via a handwritten diary. In light of these results, a refinement of the mobile app supported by a machine learning approach is planned within an international research project.

## Introduction

Allogeneic hematopoietic stem cell transplantation (HSCT) is a curative treatment for an increasing range of mostly malignant hemato-oncological diseases. Due to progress in transplant procedures, a growing number of survivors are subject to many comorbidities and late effects, which are associated both to treatment and related complications [1,2]. Among the latter, acute and chronic graft-versus-host disease (cGVHD) are the most significant, affecting survival and multidimensional aspects of quality of life [3-5]. Survivors require long-term follow-up care by highly specialized clinics, with tight communication and clinical control schemes [6]. In turn, patients' motivation and compliance are crucial factors for the success of aftercare and rehabilitation.

It is now widely accepted that teenage and young adult patients undergoing cancer treatments and HSCT have a number of informational and psychosocial needs that are frequently not addressed. These needs are practical as well as being related to counseling and coping strategies [7]. Moreover, loss of compliance has been shown among adolescent cGVHD survivors during long-term follow-up, indicating that there is an unmet need to enhance monitoring of comorbidities, graft-versus-host disease (GVHD) symptoms, and quality of life during aftercare in adolescents [8].

Currently, adolescent HSCT aftercare at the St. Anna Children’s Hospital in Vienna is based on a traditional paper diary, which carries important disadvantages that are in line with reports by others: (1) the adherence by patients is low, (2) the diaries are often not filled regularly, (3) the accuracy of symptom recording diminishes when recall periods extend (ie, recall bias), and (4) health data are entered retrospectively and only prior to the clinic visit (ie, fake compliance) [9,10]. These disadvantages may be prevented by real-time electronic symptom report (eg, via smartphone or tablet apps adding tracking abilities) [9-11]. Adolescents are frequent technology consumers and feel more comfortable with the electronic exchange of sensitive information [10]. Today, many opportunities exist to record health-related data due to the development of new technologies [12]. Real-time symptom recording may provide the possibility to detect negative health changes earlier, independent of hospital visits, providing the possibility to initiate coping strategies [13]. In summary, the use of mobile apps for symptom self-report satisfies patients’ preferences, improves compliance, and reduces errors in data collection [9,14-17]. It is well accepted that gamification approaches may enhance users’ motivation and satisfaction, along with compliance and ability for self-management [16,18].

Most mobile app publications for adolescent cancer patients have focused on pain; less data are available for HSCT patients. Generally, the majority of studies examined usability, acceptability, and adherence to app use. Although there is now a plethora of health mobile apps available [19], a review published in 2015 revealed only four pilot-tested mobile apps for young cancer patients [20]. Medical professionals are rarely included in the development of health-related software [13]. A review by Payne et al outlined the need to determine the efficacy of mobile apps [21]. To our knowledge, no study has compared the quality of medical information for the clinician when obtained via a mobile app with the information content obtained via a handwritten paper diary.

Recently, we have developed a prototype of a web-based, self-monitoring, gamified smartphone app, designed for adolescents in particular, as part of a third-party funded project—INTERACCT (Integrating Entertainment and Reaction Assessment into Child Cancer Therapy) [22]—together with academic partners from the University of Vienna, Entertainment Computing Research Group, and the University of Applied Arts Vienna, as well as the industry partner T-Systems Austria. To enhance motivation for data submission by users, the INTERACCT app was co-designed with adolescents and embedded into a serious game.

This study describes the evaluation of the INTERACCT app for tracking real-time self-reported symptoms and health status data in adolescents after HSCT in comparison with a healthy age- and gender-matched control group. As a first step on the way to test the INTERACCT app within a randomized controlled trial, we defined the high quality of medical information transfer as an indicator of efficacy. Therefore, we wanted to determine whether the quality of medical information reported via the mobile app is at least as informative for the clinician as that reported by the traditional paper diary, which has been used so far. Within this observational study, funded by a grant from the Austrian Society for Hematology and Oncology, clinicians evaluated the quality of the reported medical information and compared it with the information obtained from handwritten diaries, both from patients and from an age-matched healthy control group. We hypothesized that the mobile app provides significantly superior medical information for the clinicians than the handwritten diaries.

## Methods

### Participants

The observational, prospective INTERACCT study was conducted among HSCT survivors during regular visits at the HSCT outpatient clinic of the St. Anna Children’s Hospital. Inclusion criteria were as followed: life expectancy of more than 6 months, at least 12 years of age, and no evidence of recurrence of primary disease. Healthy students from two classes at a Viennese school were recruited on a voluntary basis as the control group. Study briefing and instructions for use were given by an HSCT clinician visiting their school. Written informed consent was taken home, completed, and collected by the involved teacher. The study was conducted in accordance with the Declaration of Helsinki and has been approved by the institutional review board of the Medical University of Vienna and the St. Anna Children’s Hospital. Furthermore, the project was reviewed and approved by Vienna’s Board of Education (*Stadtschulrat Wien*, in German). All participants and parents were informed that the individual results of the project evaluations could not be disclosed. Written informed consent was obtained from participants and parents; 52 participants—16 patients (31%) and 36 healthy control group students (69%)—between the ages of 12 and 19 years were pseudonymously enrolled. No cointerventions or training sessions were given to the participants.

Participants’ sociodemographic data were self-reported, and patients’ medical data were retrieved from medical records. Cancer treatment–associated (ie, for malignant underlying diseases) severe comorbidity was classified according to the National Cancer Institute (NCI) Common Terminology Criteria for Adverse Events (CTCAE) v3.0 [23] with a grade of 3-4. The HSCT-associated complication cGVHD was classified according the National Institutes of Health consensus criteria for cGVHD [24].

**Figure 1:**
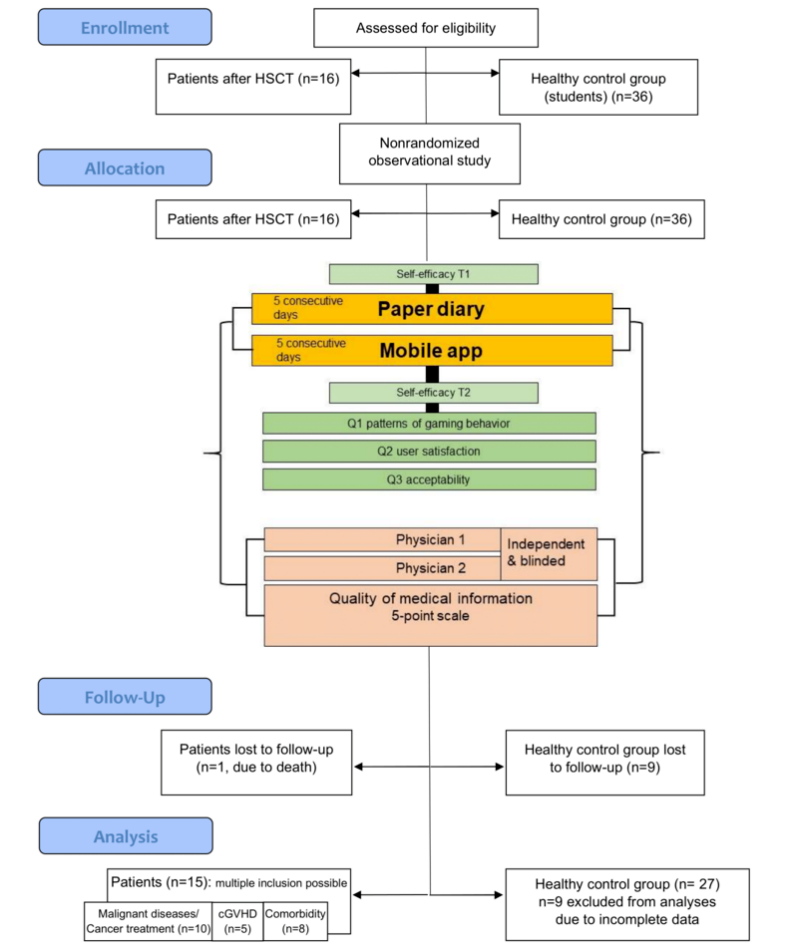
Study overview. The diagram was adapted from the CONSORT (Consolidated Standards of Reporting Trials) 2010 flow diagram (Eysenbach, 2011, and CONSORT, 2020). Malignant diseases include all malignant underlying diseases. Comorbidity is deemed severe when graded as 3-4 on the Common Terminology Criteria for Adverse Events (CTCAE). cGVHD: chronic graft-versus-host disease; HSCT: hematopoietic stem cell transplantation; Q: question; T1: time point 1 at study entry; T2: time point 2 at study termination.

Participants used the paper diary first for 5 consecutive days, from Monday to Friday, followed by 5 days using the mobile app, also from Monday to Friday, to report given health status parameters and symptoms. The study included a no-waiting-list design. An overview of this observational study is shown as a diagram adapted from the CONSORT (Consolidated Standards of Reporting Trials) 2010 flow diagram (see Figure 1) [19,25].

### Instruments

**Figure 2:**
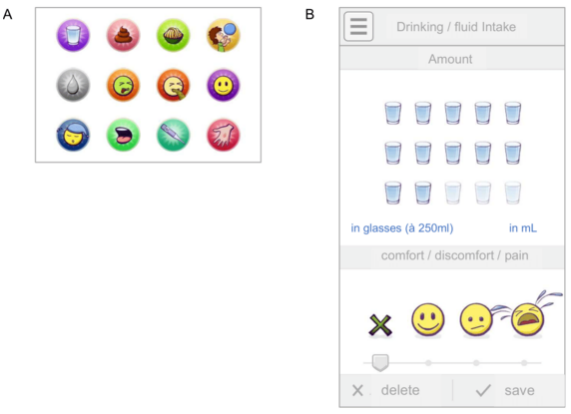
INTERACCT (Integrating Entertainment and Reaction Assessment into Child Cancer Therapy) app screenshots. A: Icons for the domains of self-reported health status—fluid and food intake (amount and difficulties); urine and stool (frequency, quality, and pain); physical exercise and play (duration in minutes); nausea and vomiting (frequency); fatigue (frequency); mouth complaints (frequency, quality, and pain); body temperature; and skin complaints (localization, frequency, and quality). B: Input for amount of fluid intake and difficulties, as an example.

The primary outcome of the study was the *quality of the self-reported medical information*, which was rated independently and blinded by two experienced HSCT physicians on a 5-point scale, ranging from 1 (low medical information content) to 5 (high medical information content). The traditional paper diary asked for seven domains to be reported on: stool frequency and quality, urinary abnormalities, nausea and vomiting, body temperature, pain, well-being, and activity—pain and well-being were scored on a 3-point scale. No further specific questions have been raised, but users had the possibility to add comments freely. The *medical information* collected in the mobile app included 12 domains. See Figure 2 for more details.

To further assess the *usability* of the measure, several aspects were investigated. For assessment of user satisfaction, an adapted version of the Game Experience Questionnaire was employed [26]; items were measured on a 3-point Likert scale, ranging from 1 (not at all) to 3 (extremely). Participants were asked to report how bored, impressed, frustrated, tired, irritated, skillful, satisfied, challenged, strained, and well they felt during the mobile app usage. App-design acceptability was assessed by participants on a 3-point scale, ranging from 1 (worst) to 3 (best), for each of the 12 health domains and symptoms (see Figure 2). Additionally, participants were asked to share suggestions for improvements of the mobile app.

For capturing patterns of gaming behavior, we designed an in-house questionnaire. Participants were asked to rate how often they play video games alone or with friends and how often they use certain devices on a 3-point Likert scale, giving a score of 1 (less than once a week), 2 (about once a week), or 3 (several times a week or daily). Finally, the average time spent, daily, on game activities was requested.

Self-efficacy of participants was assessed using a modified version of the Jerusalem Scales, in order to uncover the psychosocial background of participants. Self-efficacy questionnaires were requested at study entry (T1) and termination (T2) as outlined in the study overview [27]. The self-efficacy scales are widely used and target self-beliefs, cognitive and behavioral strategies necessary for problem solving (ie, coping), positive perception of the future (ie, optimism), as well as individuals’ appraisal of stress (ie, challenge, threat, and loss perception); items are scored on a 4-point Likert scale, ranging from 1 (strongly disagree) to 4 (strongly agree) [27].

### Technical Components

The technical components were comprised of a Unity 3D 8 app (Unity Technologies) for the participants and an ASP.NET 9 web interface (Microsoft) for the clinicians. The persistence layer was implemented through the ASP.NET back end, accessing a Microsoft SQL (Structured Query Language) database, running in a secure Windows Server 2016 environment. The connection between clients and server was established using Secure Sockets Layer (SSL). Basic authentication and cross-site request forgery tokens were used to ensure data integrity and security. The INTERACCT client is a native smartphone app for Android and iOS. The main components of the client are remote medical data entry (ie, self-reported symptom and health status) as well as the game content itself. Scoring of symptoms mostly followed a 0-3 scale and was accumulated by the back end for each day. Figure 2 shows details of the mobile app with the health status domains (A) and, as an example, the input per amount of fluid intake and difficulties, thereby (B). When completing the data entry, users were rewarded with virtual in-game currency, used to progress in the main game. The core game idea of INTERACCT is to collect avatars and complete procedurally generated levels. The levels come in different graphical settings with different characters, in order to provide variety. A detailed developmental project description, as well as design considerations and preliminary results, are described by Kayali et al, Peters et al, and Mateus-Berr et al [28-31].

### Statistical Analysis

The aim of the study was to determine whether the quality of electronically collected, self-reported medical information was comparable to that of traditional paper-and-pencil self-assessment. The participants first used the paper-and-pencil self-report form for 5 consecutive days, followed by a 5-day period using the INTERACCT app. The physician-rated quality of the self-reported medical information in both scenarios was used as *primary outcome measure*. To investigate differences in the primary outcome, one-way repeated-measures analyses of variance (ANOVAs) were calculated. Only patients with complete datasets were included in the analyses. The between-subject factor *group* was included to examine any differences between patients and students. We used *t* tests for associated samples as post hoc tests. Since, to our best knowledge, no prior studies have compared the quality of self-assessed medical information between paper-and-pencil and electronic assessments, and due to the observational nature of this pilot study, no a priori sample size calculations were feasible. Yet, to investigate whether the main results of the study are valid, a post hoc power analysis was conducted using G*Power 3.1 (Heinrich-Heine-Universität). To evaluate the interrater reliability of the physician-based evaluations, intraclass correlation coefficients (ICCs), based on a two-way mixed model and the absolute agreement, were calculated. The primary outcome measure was correlated with participants’ demographics—age ≤13 years versus ≥14 years, gender, and native language German or other—to investigate potential confounding effects. Due to the small sample sizes of these groups, descriptive methods (ie, mean and SD) were preferred over interference statistics. For descriptive correlation with patients’ demographics, subgroups were defined as follows: malignant versus nonmalignant underlying diseases, cGVHD (yes vs no), and severe comorbidity (CTCAE grade of 3-4) versus no comorbidity or mild comorbidity (CTCAE grade of 0-2) [23]. For assessment of users’ satisfaction and acceptability of the mobile app design, the median and IQR were calculated for each item.

## Results

### Participants

Only participants who used both the paper diary and the mobile app for 2 or more days and completed all questionnaires were included in the analyses. Data from 1 patient (due to death) and 9 students (due to incomplete data) had to be excluded, leaving data from 42 participants for further analyses: 15 patients, mainly after HSCT for malignant underlying diseases, and 27 healthy students (see Figure 1). Table 1 shows that the participants’ characteristics and demographic details—age, gender, and native language—were comparable (data not shown). Note that mean age was higher (data not shown) for some patient subgroups (ie, patients with malignant underlying diseases, cGVHD, and severe comorbidities).

**Table 1:** Participant characteristics.

Characteristic				Value
**Patients (N=15)**				
	Female, n (%)		8 (53)	
	Age in years, mean (SD)		14.40 (2.29)	
	Age (≥14 years), n (%)		9 (60)	
	Native language (German), n (%)		12 (80)	
	**Malignant underlying disease (n=10)**			
		Total, out of all 15 patients, n (%)	10 (67)	
		Female, n (%)	6 (60)	
		Age in years, mean (SD)	15.20 (3.97)	
		Time since HSCT^a^ in years, mean (SD)	3.22 (4.53)	
	**cGVHD^b^ (n=5)**			
		Total, out of all 15 patients, n (%)	5 (33)	
		Female, n (%)	5 (100)	
		Age in years, mean (SD)	16.20 (2.78)	
	**Comorbidity: CTCAE^c^ grade 3-4 (n=8)**			
		Total, out of all 15 patients, n (%)	8 (53)	
		Grade of 3, n (%)	5 (63)	
		Grade of 4, n (%)	3 (37)	
		Female, n (%)	5 (63)	
		Age in years, mean (SD)	16.00 (2.73)	
**Healthy control group (N=27)**				
	Female, n (%)		13 (48)	
	Age in years, mean (SD)		13.22 (1.12)	
	Native language (German), n (%)		19 (70)	

^a^HSCT: hematopoietic stem cell transplantation.

^b^cGVHD: chronic graft-versus-host disease.

^c^Classification according to the National Cancer Institute (NCI) Common Terminology Criteria for Adverse Events (CTCAE) [23].

### Analysis of Patterns of Gaming Behavior

Our results showed different patterns of gaming behavior between patients and the healthy control group. Generally, the playing frequency was found to be higher in patients than in students, with patients playing daily or at least several times a week (10/15, 67% vs 12/27, 44%, respectively). This finding was more pronounced in males, in older participants (ie, ≥14 years of age), and in participants whose native language is German.

The majority (10/15, 67%) of patients reported playing computer games alone rather than with friends (1/15, 7%). This applied particularly to the more-impaired patient groups with cGVHD and comorbidities (CTCAE grade of 3-4), of which not a single person indicated playing games with friends. In contrast, 10 out of 27 (37%) students from the control group reported playing with friends. Most of the participants spent an average of 2 hours (up to 2.65 hours) per day playing computer games. Notably, even the younger users (ie, ≤13 years of age) reported using mainly the smartphone for about 2 hours a day. The most preferred kind of personal computing device was clearly the smartphone among the majority of the users (patients: 10/15, 67%, and healthy controls: 16/27, 59%); this was mostly evident in the patient group with malignant diseases, cGVHD, and severe comorbidities (see Multimedia Appendix 1).

### Comparison of the Paper Diary With the Mobile App

#### Overview

**Figure 3:**
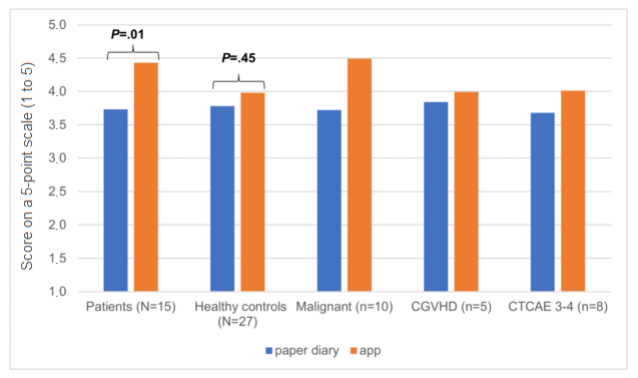
Comparison of the paper diary with the mobile app. The plot shows the clinician-reviewed quality of medical information regarding patient-reported health status on a 5-point scale, ranging from 1 (low) to 5 (high). Malignant refers to malignant underlying disease. cGVHD: chronic graft-versus-host disease; CTCAE 3-4: National Cancer Institute (NCI) Common Terminology Criteria for Adverse Events (CTCAE) grade of 3-4.

Over the entire study cohort, the clinicians rated the medical information content of the mobile app with an average of 4.14 (SD 1.14) points versus 3.77 (SD 0.91) points for the paper diary (see Figure 3). The repeated-measures ANOVA revealed a significant difference between the information content of the mobile app and the paper diary, with a medium-to-large effect size (*F*_1,40_=5.571, *P*=.02, η²=.12). Overall, results indicate that the mobile app provided significantly more information for the clinicians than the handwritten diary. Additionally, the between-subject factor group was not significant (*F*_1,40_=0.522, *P*=.47) and had no significant interaction effect (*F*_1,40_=1.807, *P*=.19). A post hoc power analysis revealed a power of β=.99 for the repeated-measure ANOVA, indicating a sufficiently powered sample for this analysis.

Post hoc *t* tests confirmed that the significant main effect was predominantly driven by the patients. The patients received significantly higher average points for the mobile app than for the paper diary (mobile app: mean 4.43, SD 1.07, vs paper diary: mean 3.73, SD 0.63, *t*_1,14_=-2.941, *P*=.01). No significant difference between the mobile app and the paper diary was found for the control group (mobile app: mean 3.98, SD 1.17, vs paper diary: mean 3.78, SD 1.05, *t*_1,26_=-0.772, *P*=.45). No correlation with user-specific characteristics, such as age, gender, and native language, was observed. A tendency toward higher information content ratings via the mobile app in patients with malignant diseases was observed (see Figure 3). However, the mobile app always delivered higher ratings for the quality of medical information than did the handwritten diary.

#### Quality of Reported Medical Information Content

The quality of the self-reported medical information was rated independently by two blinded experienced HSCT clinicians on a 5-point scale, ranging from 1 (low medical information content) to 5 (high medical information content), and revealed a high interrater reliability among them (see Figure 3). The average ICC was .867 (95% CI .733-.932, *F*_41,41_=8.530, *P*≤.001) for the paper diary and .912 (95% CI.794-.958, *F*_41,41_=14.001, *P*≤.001) for the mobile app.

#### Duration of Data Entry in Days

**Figure 4:**
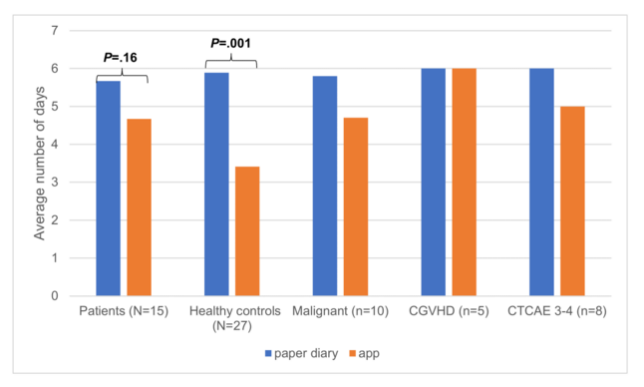
Comparison of the paper diary versus mobile app: duration in days of data entry. Malignant refers to malignant underlying disease. cGVHD: chronic graft-versus-host disease; CTCAE 3-4: National Cancer Institute (NCI) Common Terminology Criteria for Adverse Events (CTCAE) grade of 3-4.

As seen in Figure 4, patients used the paper diary more than required for an average of 5.67 (SD 1.23) days. The mobile app was used by this group for an average of 4.67 (SD 2.53) days, but this difference was not significant (*t*_1,14_=1.479, *P*=.16). In contrast, the control group used the diary as long as the patients did, but the mobile app was used for a significantly shorter time than was the paper diary (mean 5.89 days, SD 1.31, vs mean 3.41 days, SD 1.69, respectively; *t*_1,26_=6.138, *P*≤.001). Correlation analysis with users’ demographics showed that the mobile app, but not the paper diary, was used significantly longer by the adolescent group (≥14 years: 4.57 days, vs ≤13 years: 3.14 days, *t*_1,40_=-2.331, *P*=.03) and females (4.76 days for the females vs 2.95 days for the males, *t*_1,40_=3.082, *P*=.004).

The descriptive correlation with patient subgroup characteristics showed a positive correlation (ie, longer duration of data entry) with cGVHD (cGVHD: 6.00 days, vs no cGVHD: 4.00 days) and comorbidity (CTCAE 3-4: 5.00 days, vs CTCAE 0-2: 4.10 days).

### General User Satisfaction and Acceptability of the Mobile App Design

User satisfaction for the mobile app was high across all groups. Table 2 shows the median score and the IQR for all the user satisfaction items.

**Table 2:** User satisfaction of the mobile app.

User satisfaction item	Score^a^, median (IQR)
I was bored	2.00 (2.00)
I was impressed	2.00 (2.00)
I felt frustrated	1.00 (1.00)
I found it tiring	1.00 (1.00)
I was irritated	1.00 (1.00)
I felt skillful	2.00 (2.00)
I was satisfied	3.00 (3.00)
I felt challenged	1.00 (1.00)
I had to put a lot of effort into playing	1.00 (1.00)
I felt good	3.00 (3.00)

^a^Scores range from 1 (not at all) to 3 (extremely).

In particular, we found that most participants (minimum 25/42, 60%, maximum 40/42, 95%) felt hardly frustrated, tired, irritated, or strained. On average, more than half of the participants stated that they were very satisfied during the mobile app usage, were hardly challenged, and felt good (see Multimedia Appendix 2).

Patient subgroups with malignant diseases, cGVHD, and CTCAE grade 3-4 representing the most experienced patients indicated that they were a little bored with the mobile app (see Multimedia Appendix 3). This finding corresponds with the result that relatively more patients (13/15, 87%) felt hardly challenged by the mobile app compared to the healthy control group (15/27, 56%). However, nearly half of the patients (7/15, 46%) were impressed.

Additionally, the acceptability of the app design on a 3-point Likert scale—1 (negative rating), 2 (neutral rating), or 3 (positive rating)—for each of the 12 domains revealed a median score of 3 (IQR 3). In summary, the app rating was encouraging, and there was no evidence that the diary was preferred to the mobile app.

All users were asked for suggestions for intervention and app improvement. Features desired by the users included (1) the insertion of comments and functions, such as *scrolling* within the app, (2) the overview of the entered data, making visible the progress or decline they have experienced, (3) the development of a more colorful design, (4) the implementation of captions for the symbols, (5) the integration of the fitness domains into the physical exercise domain, aiming for reduced parameters that they are asked about, and (6) a broader variety of health games.

### Self-Efficacy

No significant differences in self-efficacy between user cohorts and time points (T1 vs T2) were observed using the Welch *t* test. Descriptive comparison of patient subgroups revealed that the most impaired patients, with cGVHD and comorbidity CTCAE grade 3-4, reported lower scores for optimism, coping, and general self-efficacy, while reporting higher scores for loss and threat perception, in comparison to other patients. All findings remained stable over the period of the study (data not shown).

We summarized several findings of our study, which may be specific for the target group of impaired adolescent patients after cancer and HSCT, and created a case vignette for further use by developers and researchers (see Multimedia Appendix 4).

## Discussion

### Principal Findings

Recently, we have developed a web-based, self-monitoring mobile app for adolescent patients after cancer treatment and HSCT, within the multidisciplinary research project INTERACCT, at the interface of clinical research, design, and information technology [28-30]. This study describes the multidimensional testing of the co-designed INTERACCT app for tracking real-time self-reported symptoms and health status data in patients compared with a healthy age- and gender-matched control group. Our results show that mobile app use, when compared to the traditional paper diary, resulted in significantly improved quality of medical information for the clinicians. The mobile app outperformed the paper-and-pen assessment, particularly within the patient cohort; this was accentuated in more-impaired patients with treatment-associated complications, suggesting that the app is suitable for the group it was developed for. In accordance with the highly rated usability and acceptability, our results indicate that adolescents, after cancer treatment and HSCT, would use a real-time, self-monitoring smartphone app for tracking symptoms and changes in their health status, resulting in improved patient-physician communication.

As data submission through the INTERACCT app was embedded into a serious game, we also analyzed patterns of gaming habits and preferences for electronic devices; clearly, the smartphone is the appropriate device for our possibly chronically ill target group. Not surprisingly, the identified patterns of gaming habits may reflect the living situations of HSCT patients, who are often not participating in age-appropriate activities with peers. Our data suggest that, especially for adolescent patients with treatment-associated complications who are living a more isolated life, the implementation of a smartphone-based symptom-reporting tool seems appropriate, as described previously for cancer patients [18-20].

In addition to published app studies in cancer patients, which mostly described the development, implementation, and usability testing, we hypothesized that the quality of the medical information, which was submitted through a serious game, would be superior to the medical information collected via handwritten diary [32,33]. Our results confirm this hypothesis significantly for the patient group (mobile app: 4.43 points, vs paper-based diary: 3.73 points, *P*=.01) and the finding is even more significant in cancer patients. The superiority of the mobile app is evident regarding the duration of symptom recording within the group of impaired adolescents after HSCT: symptom report via mobile app was significantly longer in patients over 13 years of age, female, with cGVHD, and with comorbidities with CTCAE grade 3-4 (cGVHD: 6.00 days, vs no cGVHD: 4.00 days; CTCAE 3-4: 5.00 days, vs CTCAE 0-2: 4.10 days). This may be due to several reasons: (1) symptom assessment 24/7 makes recording possible when the health problem arises, overcoming the problem of the recall period [9], and (2) motivation may have been enhanced by the serious game [18]. Fortier et al developed Pain Buddy for young cancer patients, providing an animated avatar, gamification components, and remote symptom monitoring, revealing high user satisfaction (n=12) while keeping track of their pain [34]. Baggott et al first published the results of utility testing of an electronic diary among 10 adolescent cancer patients, which not only reported pain but additionally reported nausea, vomiting, fatigue, sleep, and mood, and showed high adherence and reported benefit [10].

One may speculate that patients with more serious symptoms show better adherence comparable to diabetes patients [35]. Accordingly, the patients with malignant diseases, comprised of experienced patients without suffering from severe complications, scored better regarding the quality of medical information, even though they used the app for a shorter duration. Similarly, Rodgers et al reported an extensive decrease of app usage over time—the app EAT! assists in self-management of eating difficulties—in 16 HSCT patients [36]. Regarding the lower results of the mobile app usage within the control group, we speculate that the lack of symptoms hampers the conscientious documentation of health parameters; finally, the mobile app does not allow retrospective filling. Indeed, when we went back to the paper diaries of the control group, we found evidence for copy and paste in about 50% of paper diaries, comparable to the results by Stone et al (ie, fake compliance) [9].

In line with many published data, the usability and acceptance of the mobile app and its design was very positive and there was no evidence that the diary was preferred to the mobile app [18,20,37]. As indicated by Crosby et al, the participation of a multidisciplinary team combining experts of clinical research, design, and information technology may have been beneficial [38]. High compliance rates and acceptability in adolescent cancer patients have been reported by the use of Pain Squad as an iPhone app [16]. Subsequently, the group was able to show construct validity and reliability of the app [39] but, recently, the implementation in a natural setting has outlined important challenges [40].

The descriptive results of the self-efficacy evaluation stress the importance of the implementation of a mobile app for improved patient-physician communication within the vulnerable HSCT patient group suffering from severe comorbidities and cGVHD.

### Limitations

The main limitation of this study is related to the small sample size making statistical analyses of the subgroups impossible. Another drawback is that the intervention periods were quite short. Even though we used an age- and gender-matched control group, it seems very likely that the patients were more experienced regarding the self-report of symptoms. Additionally, the criteria for selecting the health domains with various symptoms for the INTERACCT app were partly empirically based and the number was perceived as too high—12 mobile app domains versus seven paper diary domains—by most users. Retrospectively, the time points of testing the self-efficacy seem to have been too close.

Although there is a myriad of reports about utility testing in mobile apps tracking various symptoms in cancer patients, Wesley and Fizur comprehensively outline the limitations, such as small sample numbers, poor correlation with sociodemographic parameters, and short intervention periods, and they highlight further research efforts [20].

Nevertheless, we have learned important lessons: patients after cancer treatment and HSCT represent a highly experienced target group regarding smartphone-based apps and health games. This has to be considered for further refinement as does the implementation of health games where the competition from thousands of available products is quite high. Pereira et al reported that gamification elements enhance users’ motivation initially, but the interest declines over time [18]. The number of self-reported health domains requires careful consideration because of the wide variation in patients’ abilities to maintain attention to tasks. Hence, it might be useful to reduce the number of items in the app and to minimize the associated exertion. The high usability of the mobile app was not sufficient enough to foster sustained health data entry of our comorbid patients in the long term [41]. In addition to usability, illness experience, information technology infrastructure, emotional activation, degree of burden caused by the app, and relevance of symptom monitoring are important factors for patient motivation [42]. The less data that users are asked to enter, the greater their level of adherence in general [43]. Adaptive individual patient profiles, aiming for collection of relevant health data only, are warranted.

### Implications of the Pilot Study

Our results have various implications: real-time assessment of symptom and health status changes may improve understanding of treatment-associated complications promoting enhanced supportive care. The model of real-time assessment described in this study can be used in both clinical and research contexts to evaluate the effectiveness of diagnosis and treatment. Ideally, it would move the field of clinical HSCT-aftercare research forward with identifying prodromes of complications in the future. INTERACCT already includes a web interface for clinicians to observe the submitted data, but its evaluation was not in the scope of this study. Plans are in place to test the refined mobile app within a multicenter randomized controlled study. We are planning not only to refine the INTERACCT app but, additionally, to apply machine learning algorithms aiming for implementation of adaptive individual patient profiles [44], thus enabling the mobile app to collect relevant health data and symptoms only.

### Conclusions

In this study, we found support for its main hypothesis, that the quality of patient-reported medical information submitted via the INTERACCT app embedded in a serious game is superior to that from the traditional handwritten diary. The statistical results confirm this hypothesis. This is especially important for the target group of adolescent patients after cancer treatment and HSCT with severe comorbidities, where the results were even more conclusive than in the healthy control group. With this in mind, and given the scope for improvement in this relatively new research area, we plan to refine the use of the mobile app by considering machine learning approaches within an international multidisciplinary research project.

## Appendix

Multimedia Appendix 1 Participant preferences for electronic devices. Malignant refers to malignant underlying disease. cGVHD: chronic graft-versus-host disease; CTCAE 3-4: National Cancer Institute (NCI) Common Terminology Criteria for Adverse Events (CTCAE) grade of 3-4.

Multimedia Appendix 2 User satisfaction: percentage of users who felt very impressed, very skillful, very satisfied, hardly challenged, and very well.

Multimedia Appendix 3 User satisfaction: percentage of users who felt hardly bored, hardly frustrated, hardly tired, hardly irritated, and hardly strained.

Multimedia Appendix 4 Case vignette: specifics of impaired adolescent patients after cancer and hematopoietic stem cell transplantation (HSCT).

## Abbreviations

ANOVA: analysis of variance
cGVHD: chronic graft-versus-host disease
CONSORT: Consolidated Standards of Reporting Trials
CTCAE: Common Terminology Criteria for Adverse Events
GVHD: graft-versus-host disease
HSCT: hematopoietic stem cell transplantation
ICC: intraclass correlation coefficient
INTERACCT: Integrating Entertainment and Reaction Assessment into Child Cancer Therapy
NCI: National Cancer Institute
OeGHO: Die Österreichische Gesellschaft für Hämatologie & Medizinische Onkologie
SQL: Structured Query Language
SSL: Secure Sockets Layer
T1: study entry
T2: study termination

## References

[1] Parsons SK, Phipps S, Sung L, Baker KS, Pulsipher MA, Ness KK (2012). NCI, NHLBI/PBMTC First International Conference on Late Effects after Pediatric Hematopoietic Cell Transplantation: Health-related quality of life, functional, and neurocognitive outcomes. Biol Blood Marrow Transplant.

[2] Lawitschka A, Peters C (2018). Long-term effects of myeloablative allogeneic hematopoietic stem cell transplantation in pediatric patients with acute lymphoblastic leukemia. Curr Oncol Rep.

[3] Fraser CJ, Bhatia S, Ness K, Carter A, Francisco L, Arora M, Parker P, Forman S, Weisdorf D, Gurney JG, Baker KS (2006). Impact of chronic graft-versus-host disease on the health status of hematopoietic cell transplantation survivors: A report from the Bone Marrow Transplant Survivor Study. Blood.

[4] Sun C, Francisco L, Kawashima T, Leisenring W, Robison LL, Baker KS, Weisdorf DJ, Forman SJ, Bhatia S (2010). Prevalence and predictors of chronic health conditions after hematopoietic cell transplantation: A report from the Bone Marrow Transplant Survivor Study. Blood.

[5] Lawitschka A, Güclü ED, Varni JW, Putz M, Wolff D, Pavletic S, Greinix H, Peters C, Felder-Puig R (2014). Health-related quality of life in pediatric patients after allogeneic SCT: Development of the PedsQL Stem Cell Transplant module and results of a pilot study. Bone Marrow Transplant.

[6] Dietz AC, Duncan CN, Alter BP, Bresters D, Cowan MJ, Notarangelo L, Rosenberg PS, Shenoy S, Skinner R, Walters MC, Wagner J, Baker KS, Pulsipher MA (2017). The Second Pediatric Blood and Marrow Transplant Consortium International Consensus Conference on Late Effects after Pediatric Hematopoietic Cell Transplantation: Defining the Unique Late Effects of Children Undergoing Hematopoietic Cell Transplantation for Immune Deficiencies, Inherited Marrow Failure Disorders, and Hemoglobinopathies. Biol Blood Marrow Transplant.

[7] Husson O, Huijgens PC, van der Graaf WTA (2018). Psychosocial challenges and health-related quality of life of adolescents and young adults with hematologic malignancies. Blood.

[8] Hilgendorf I, Greinix H, Halter JP, Lawitschka A, Bertz H, Wolff D (2015). Long-term follow-up after allogeneic stem cell transplantation. Dtsch Arztebl Int.

[9] Stone AA, Shiffman S, Schwartz JE, Broderick JE, Hufford MR (2003). Patient compliance with paper and electronic diaries. Control Clin Trials.

[10] Baggott C, Gibson F, Coll B, Kletter R, Zeltzer P, Miaskowski C (2012). Initial evaluation of an electronic symptom diary for adolescents with cancer. JMIR Res Protoc.

[11] Elliot DL, Lindemulder SJ, Goldberg L, Stadler DD, Smith J (2013). Health promotion for adolescent childhood leukemia survivors: Building on prevention science and eHealth. Pediatr Blood Cancer.

[12] Wilcox AB, Gallagher KD, Boden-Albala B, Bakken SR (2012). Research data collection methods: From paper to tablet computers. Med Care.

[13] Lalloo C, Jibb LA, Rivera J, Agarwal A, Stinson JN (2015). "There's a pain app for that": Review of patient-targeted smartphone applications for pain management. Clin J Pain.

[14] Dale O, Hagen KB (2007). Despite technical problems personal digital assistants outperform pen and paper when collecting patient diary data. J Clin Epidemiol.

[15] Marceau LD, Link C, Jamison RN, Carolan S (2007). Electronic diaries as a tool to improve pain management: Is there any evidence?. Pain Med.

[16] Stinson JN, Jibb LA, Nguyen C, Nathan PC, Maloney AM, Dupuis LL, Gerstle JT, Alman B, Hopyan S, Strahlendorf C, Portwine C, Johnston DL, Orr M (2013). Development and testing of a multidimensional iPhone pain assessment application for adolescents with cancer. J Med Internet Res.

[17] Lewis TL, Boissaud-Cooke MA, Aungst TD, Eysenbach G (2014). Consensus on use of the term "app" versus "application" for reporting of mHealth research. J Med Internet Res.

[18] Pereira E, Duarte E, Rebelo F, Noriega P (2014). A review of gamification for health-related contexts. Proceedings of the International Conference of Design, User Experience, and Usability (DUXU 2014).

[19] Eysenbach G, CONSORT-EHEALTH Group (2011). CONSORT-EHEALTH: Improving and standardizing evaluation reports of web-based and mobile health interventions. J Med Internet Res.

[20] Wesley KM, Fizur PJ (2015). A review of mobile applications to help adolescent and young adult cancer patients. Adolesc Health Med Ther.

[21] Payne HE, Lister C, West JH, Bernhardt JM (2015). Behavioral functionality of mobile apps in health interventions: A systematic review of the literature. JMIR Mhealth Uhealth.

[22] INTERACCT.

[23] The National Cancer Institute (NCI) of the National Institutes of Health (NIH) (2017). Common Terminology Criteria for Adverse Events (CTCAE), Version 5.0.

[24] Filipovich AH, Weisdorf D, Pavletic S, Socie G, Wingard JR, Lee SJ, Martin P, Chien J, Przepiorka D, Couriel D, Cowen EW, Dinndorf P, Farrell A, Hartzman R, Henslee-Downey J, Jacobsohn D, McDonald G, Mittleman B, Rizzo JD, Robinson M, Schubert M, Schultz K, Shulman H, Turner M, Vogelsang G, Flowers MED (2005). National Institutes of Health consensus development project on criteria for clinical trials in chronic graft-versus-host disease: I. Diagnosis and staging working group report. Biol Blood Marrow Transplant.

[25] CONSORT.

[26] Poels K, de Kort YAW, IJsselsteijn WA (2007). D3.3: Game Experience Questionnaire: Development of a Self-Report Measure to Assess the Psychological Impact of Digital Games.

[27] Schwarzer R, Jerusalem M (1999). Skalen zur Erfassung von Lehrer- und Schülermerkmalen.

[28] Kayali F, Silbernagl M, Peters K, Mateus-Berr R, Reithofer A, Martinek D, Lawitschka A, Hlavacs H (2016). Design considerations for a serious game for children after hematopoietic stem cell transplantation. Entertain Comput.

[29] Peters K, Kayali F, Reithofer A, Wölfle R, Mateus-Berr R, Kuczwara J, Lehner Z, Lawitschka A, Brunmaier B, Martinek D, Silbernagl M, Hlavacs H (2015). Serious game scores as health condition indicator for cancer patients. Stud Health Technol Inform.

[30] Mateus-Berr R (2015). Co-designing avatars for children with cancer. Proceedings of the 3rd International Conference for Design Education Researchers (LearnXDesign).

[31] Peters K, Buehrer S, Silbernagl M, Kayali F, Hlavacs H, Lawitschka A (2019). Evaluation of informative content of health data submitted through a mobile serious game. Proceedings of the First IFIP TC 14 Joint International Conference on Entertainment Computing and Serious Games.

[32] Dupuis LL, Cook S, Robinson PD, Tomlinson D, Vettese E, Sung L (2019). Optimizing symptom control in children and adolescents with cancer. Pediatr Res.

[33] Osborn J, Ajakaiye A, Cooksley T, Subbe CP (2020). Do mHealth applications improve clinical outcomes of patients with cancer? A critical appraisal of the peer-reviewed literature. Support Care Cancer.

[34] Fortier MA, Chung WW, Martinez A, Gago-Masague S, Sender L (2016). Pain buddy: A novel use of mHealth in the management of children's cancer pain. Comput Biol Med.

[35] Baron J, McBain H, Newman S (2012). The impact of mobile monitoring technologies on glycosylated hemoglobin in diabetes: A systematic review. J Diabetes Sci Technol.

[36] Rodgers CC, Krance R, Street RL, Hockenberry MJ (2013). Feasibility of a symptom management intervention for adolescents recovering from a hematopoietic stem cell transplant. Cancer Nurs.

[37] Dupuis LL, Ethier M, Tomlinson D, Hesser T, Sung L (2012). A systematic review of symptom assessment scales in children with cancer. BMC Cancer.

[38] Crosby LE, Ware RE, Goldstein A, Walton A, Joffe NE, Vogel C, Britto MT (2017). Development and evaluation of iManage: A self-management app co-designed by adolescents with sickle cell disease. Pediatr Blood Cancer.

[39] Stinson JN, Jibb LA, Nguyen C, Nathan PC, Maloney AM, Dupuis LL, Gerstle JT, Hopyan S, Alman BA, Strahlendorf C, Portwine C, Johnston DL (2015). Construct validity and reliability of a real-time multidimensional smartphone app to assess pain in children and adolescents with cancer. Pain.

[40] Tutelman PR, Chambers CT, Stinson JN, Parker JA, Barwick M, Witteman HO, Jibb L, Stinson HC, Fernandez CV, Nathan PC, Campbell F, Irwin K (2018). The implementation effectiveness of a freely available pediatric cancer pain assessment app: A pilot implementation study. JMIR Cancer.

[41] Scott AR, Alore EA, Naik AD, Berger DH, Suliburk JW (2017). Mixed-methods analysis of factors impacting use of a postoperative mHealth app. JMIR Mhealth Uhealth.

[42] Cohen DJ, Keller SR, Hayes GR, Dorr DA, Ash JS, Sittig DF (2015). Developing a model for understanding patient collection of observations of daily living: A qualitative meta-synthesis of the Project HealthDesign Program. Pers Ubiquitous Comput.

[43] Morren M, van Dulmen S, Ouwerkerk J, Bensing J (2009). Compliance with momentary pain measurement using electronic diaries: A systematic review. Eur J Pain.

[44] Kamdar MM, Centi AJ, Fischer N, Jethwani K (2018). A randomized controlled trial of a novel artificial-intelligence based smartphone application to optimize the management of cancer-related pain. J Clin Oncol.

