# Supplementary figures and images for: A Web-Based Mobile App (INTERACCT App) for Adolescents Undergoing Cancer and Hematopoietic Stem Cell Transplantation Aftercare to Improve the Quality of Medical Information for Clinicians: Observational Study
Source: JMIR Mhealth Uhealth. 2020 Jun 30;8(6):e18781. doi: 10.2196/18781 (PMC7367529; doi:10.2196/18781)

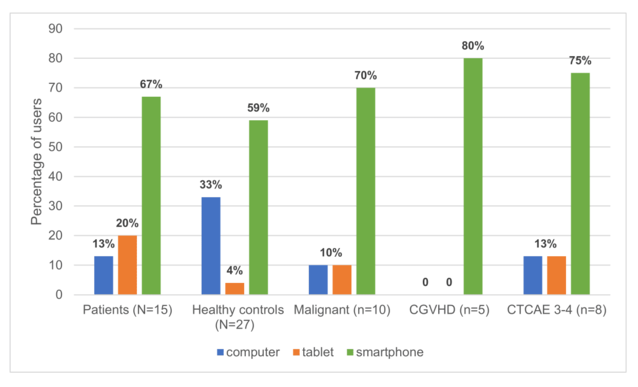

Supplement: Multimedia Appendix 1 [file mhealth_v8i6e18781_app1.png]

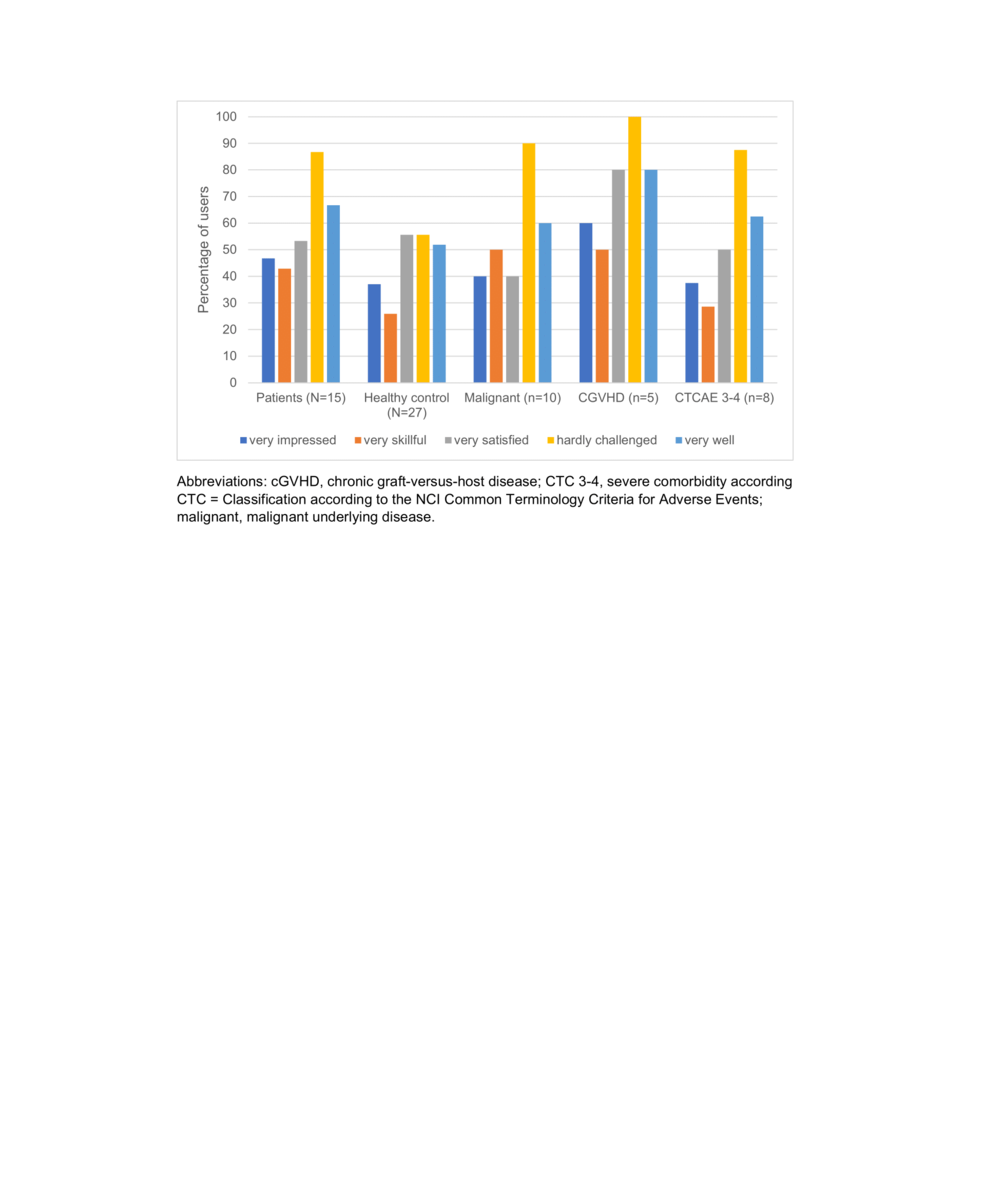

Supplement: Multimedia Appendix 2 [file mhealth_v8i6e18781_app2.png]

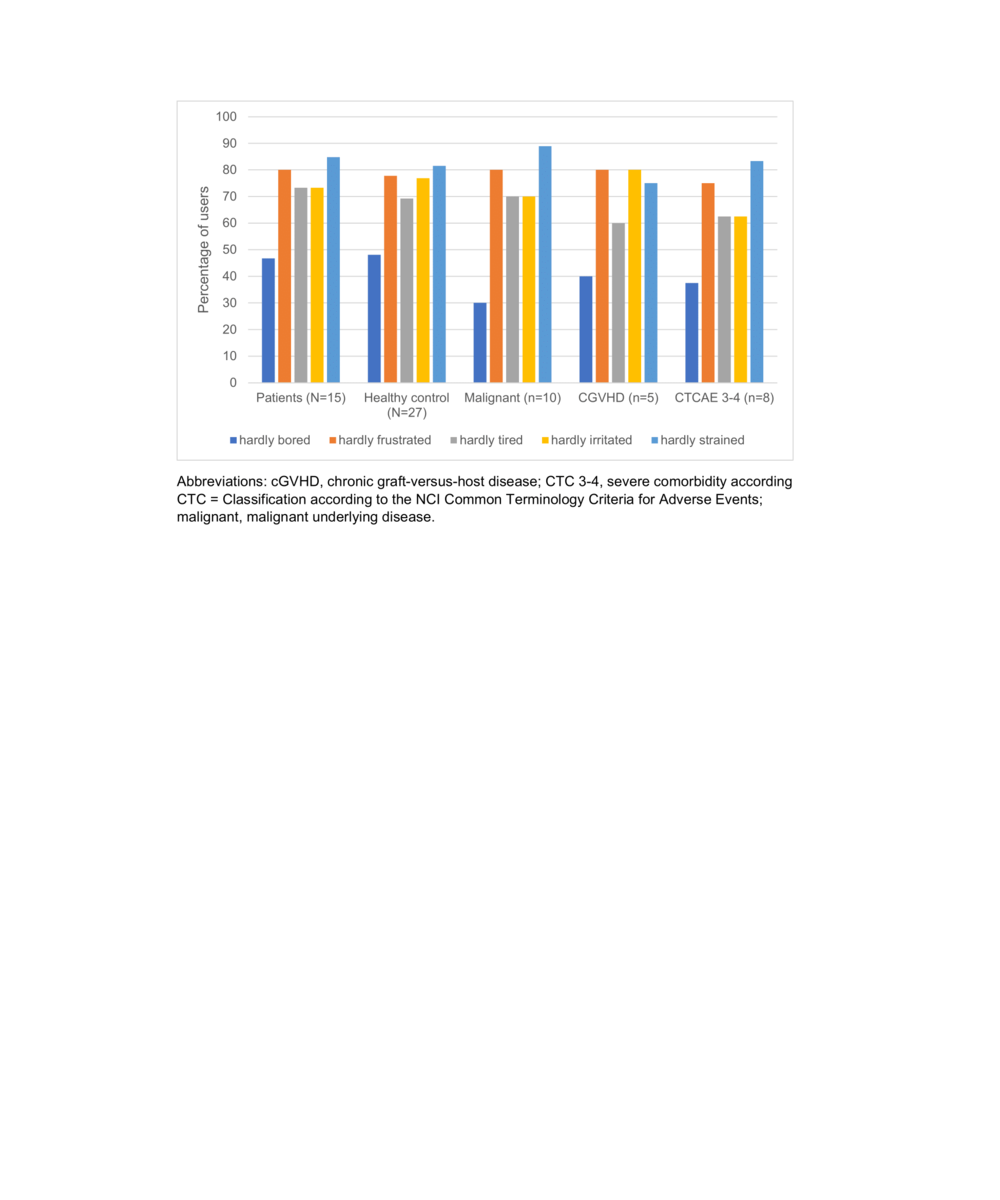

Supplement: Multimedia Appendix 3 [file mhealth_v8i6e18781_app3.png]
