# Supplementary material for: A Web-Based Mobile App (INTERACCT App) for Adolescents Undergoing Cancer and Hematopoietic Stem Cell Transplantation Aftercare to Improve the Quality of Medical Information for Clinicians: Observational Study
Source: JMIR Mhealth Uhealth. 2020 Jun 30;8(6):e18781. doi: 10.2196/18781 (PMC7367529; doi:10.2196/18781)
Supplement: Multimedia Appendix 4 [file mhealth_v8i6e18781_app4.pdf]

Multimedia Appendix 3: Case vignette: specifics of impaired adolescent patients after cancer and HSCT

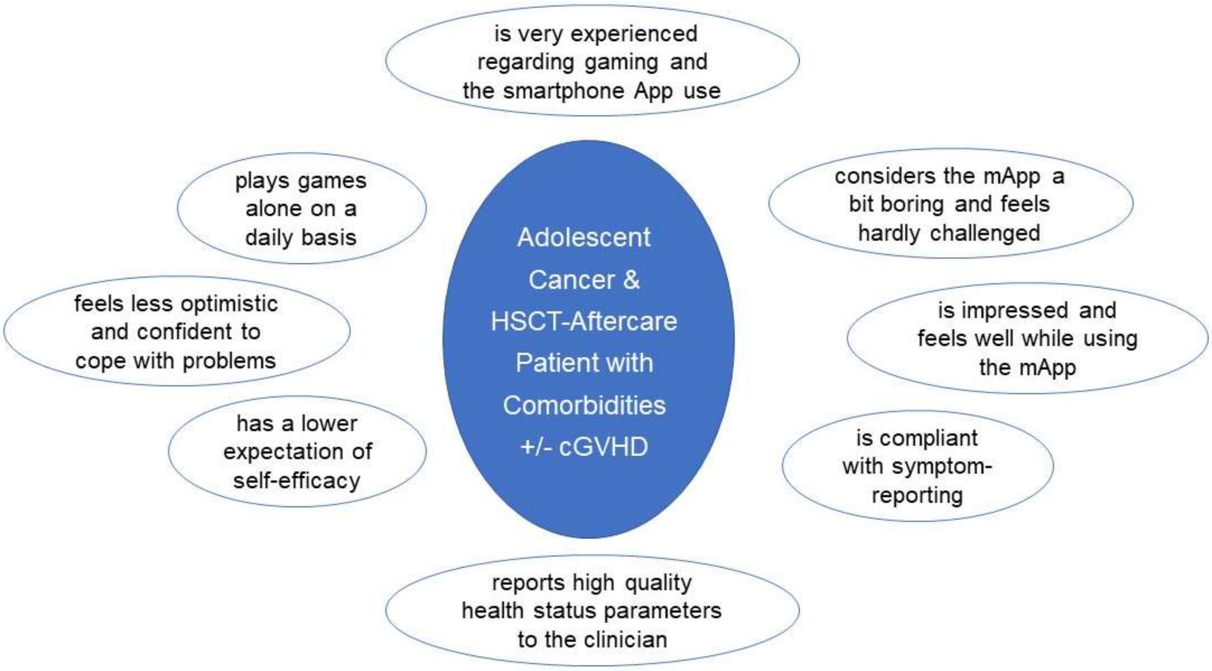

Abbreviations: cGVHD, chronic graft-versus-host-disease; HSCT, hematopoietic stem cell transplantation; mApp, mobile application.
